# Analyzing the use of videoconference by and for older adults in nursing homes: an interdisciplinary approach to learn from the pandemic

**DOI:** 10.3389/fpsyg.2023.1154657

**Published:** 2023-05-05

**Authors:** Céline Racin, Raphaël Minjard, Christophe Humbert, Vivien Braccini, Fabien Capelli, Cédric Sueur, Célia Lemaire

**Affiliations:** ^1^Centre de Recherche en Psychopathologie et Psychologie Clinique (CRPPC, EA 653), Institut de psychologie, Université Lumière Lyon 2, Lyon, France; ^2^PSInstitut, Strasbourg, France; ^3^Laboratoire interdisciplinaire en études culturelles (LinCS, UMR 7069), Université de Strasbourg, Strasbourg, France; ^4^Laboratoire Interuniversitaire des Sciences de l'Education et de la Communication (LISEC, UR 2310), Université de Strasbourg, Strasbourg, France; ^5^Institut pluridisciplinaire Hubert Curien (IPHC, UMR 7178), Centre national de la recherche scientifique (CNRS), Université de Strasbourg, Strasbourg, France; ^6^Anthropolab, Ethics (EA 7446), Université Catholique de Lille, Lille, France; ^7^Magellan (EA 3713), iaelyon, Université Jean Moulin Lyon 3, Lyon, France; ^8^Faculté des sciences de l’administration, Université Laval, Québec, QC, Canada

**Keywords:** nursing home, older adults, videoconference, COVID-19, mediation, play, consent, interdisciplinarity

## Abstract

**Introduction:**

During the Covid-19 pandemic and the resulting visitation restrictions, digital tools were used in many nursing homes in France to allow the older adults and their relatives to maintain social contact via videoconferencing. This article adopts an interdisciplinary approach to analyze the processes that affect the use of digital technologies.

**Methods:**

Drawing on the concept of “mediation,” it seeks to shed light on how individuals embrace these tools in a relational situation. The interviews and observations undertaken among residents, their relatives, professionals, and the management head of seven nursing homes in 2021, make it possible to outline the different forms of practices and uses and to identify the factors leading to the variations observed.

**Results:**

While the key objective of these technical and technological tools is to compensate – on a functional level – for the communication problems and the isolation of individuals in order to promote residents’ “quality of life” by maintaining “social contact,” our study reveals that these tools’ uses and practices largely differ. It also shows considerable inequalities in terms of residents’ acquisition of subjective feelings of ownership of the tools. These are never attributed to isolated physical, cognitive, psychic, and social difficulties, but are influenced by specific organizational, interactional, and psychic configurations. Some of the structures analyzed revealed situations in which mediation failed, occasionally exposing the risk associated with seeking “ties at all costs,” or revealing a disturbing strangeness when residents were placed in front of screens. Some configurations, however, showed that it was possible to set up an intermediate space for the experience to unfold, which in turn opened up a space where individuals, groups, and institutions could experiment, allowing them to develop subjective feelings of ownership of this experience.

**Discussion:**

This article discusses how the configurations that failed to promote the mediation process reveal the need to assess the representations of care and assistance in the relationships between older adults, their loved ones, and nursing home professionals. Indeed, in certain situations, the use of videoconferencing, while seeking to produce a positive effect, risks displacing and increasing the effects of the “negative” associated with dependency, which may worsen individuals’ difficulties within nursing homes. The risks associated with the failure to take into account residents’ requests and consent explain why it is important to discuss how certain uses of digital tools may renew the dilemma between concerns for protection, on the one hand, and respect for autonomy on the other.

## 1. Introduction: the context in which digital tools were used in nursing homes in France during the COVID-19 pandemic

During the COVID-19 pandemic in France, each nursing home sought to implement the national guidelines issued by the government from the first wave in March 2020. These nursing homes notably insisted on the suspension of visits and of residents’ collective or individual temporary outings. New admissions were also put on hold. These recommendations meant that professionals began to prioritize interventions based on whether or not they were perceived as “indispensable” for residents’ health, and those interventions judged to be indispensable were maintained in compliance with protective measures. Concerns about the isolation of residents led to the recommendations to create a space dedicated to private communications within the institution and to provide slots for making calls, except when residents’ conditions did not allow motion. In the latter case, videoconferencing tools were recommended. Only a few exceptional situations (acute somatic or psychic decompensation, end-of-life situations) permitted the rules to be relaxed.

In this context, the CovidEhpad study (Balard et al., 2021) shows that the lockdown period was experienced in a largely heterogeneous manner which took contrasting forms depending on the modes of living in nursing homes as experienced by each resident, on the occupational or relational resources that the nursing homes succeeded in using, and on the residents’ past. Moreover, while the COVID-19 pandemic limited the freedom of all French citizens, this constraint was even greater among nursing home residents, many of whom had multiple pathologies, coupled with cognitive impairments in some cases. Indeed, little attention was paid to residents’ rights and ability to question this restriction of liberty (which is a constitutional right), to their self-determination, and to their consent to lockdown procedures in their nursing homes. These restriction measures were often linked to the degree of contamination in the institution. Moreover, radical measures were occasionally adopted locally in a context where the national framework remained vague and gave insufficient consideration to respect for the fundamental rights of nursing home residents (Defendeur of Rights, 2021). Internal inequalities also emerged between the residents, and while doors had to remain open in some nursing homes, they were allowed to stay closed in others. Some residents were also allowed to move around, for instance by going into the garden or up to the institution’s doorstep.

To allow older adults and their loved ones to maintain ties via video-calls during the visitation restrictions, many French departments decided to distribute digital tablets in those nursing homes that lacked these tools. However, the dynamics of digital innovation in nursing homes still encounter great difficulties and have sometimes shown inconclusive results (Gaglio, 2018). For instance, many institutions – including those that participated in this study and irrespective of whether they had tablets before or during the pandemic – had no wifi connection and were able to “knock something together” by sharing the connection from professionals’ smartphones. Moreover, most of those institutions that already had the equipment had not necessarily developed a genuinely digital culture which would have helped to promote social ties through these tools. Digital technologies were therefore primarily used by the social coordinators to propose activities within the institution.

During the Covid-19 pandemic and the associated visitation restrictions, many nursing homes in France opted for digital tools to support remote social contact between residents and their relatives. Initially, these tools sought to compensate, on a functional level at least, for individuals’ inability to communicate – and/or for their isolation – with a view to promoting a “good enough” quality of life and to maintaining social ties. Their implementation, however, occurred in a context where major social changes have been gradually unfolding, changes of which the pandemic was simply an accelerator.

First, the Covid-19 pandemic, and the health measures implemented to address it, transformed individuals’ social experiences: they undermined the conditions of stability and security in which social contacts were habitually made, they redefined the meaning of so-called “essential” activities, and they revived the dilemma of protection versus freedom with regard to health threats, in a context that privileges the “supposedly fragile” status of older adults (Sizoo et al., 2020; Gzil, 2021; Rosier and Hecketsweiler, 2021; Racin and Rocard, 2022).

Second, the increase in the number of digital tools proposed in nursing homes during this unprecedented period was also associated with the digital transformation of healthcare (Naik et al., 2022) and, more generally, with the rapid digitization of today’s society. These tools are now widely used to maintain remote relationships among people under the age of 80, irrespective of whether they live at home or in nursing homes (Martins Van Jaarsveld, 2020). Multiple factors are thought to be behind the unequal access to these digital tools, such as the individual characteristics of the older adults, the tools’ specific features, and the support one receives to enable them to embrace these tools (Mubarak and Suomi, 2022). However, rather than reduce these inequalities, the key objective of the unprecedented digital offer proposed in nursing homes during the pandemic was to address the risk of social isolation among the older adults (Chung et al., 2020).

In this context, based on a study of digital and organizational innovations seeking to promote social ties in nursing homes in the wake of the COVID-19 pandemic (Innovehpad), the following questions arise: what are the organizational, interactional, and psychic conditions that allow individuals and groups of professionals to use and embrace these digital tools? With what objectives in mind? Indeed, while these tools tend to reduce the inequalities associated with institutionalization, notably in terms of social isolation, they may give rise to, or reinforce, other types of inequality if one fails to consider whether or not they can be embraced, and the impact of their use or non-use.

## 2. Literature review: digital realities of older people in long-term care facilities during the visitation restrictions associated with the Covid-19 pandemic period

### 2.1. Remote social contacts in the fight against social isolation of older adults

The research studies on remote social contact among nursing home residents, which have emerged over the past 20 years, have primarily relied on semi-experimental approaches. These approaches suggest that a relationship exists between the social isolation of older adults and the increased risk of mortality and morbidity, particularly in terms of depressive syndromes (Krishnan et al., 1998; Seeman, 2000; Hybels et al., 2001). This isolation is largely associated with the fact that visits tend to decrease after institutionalization Tsai and Tsai (2015) as well as with the fact that some relatives are themselves very old, have mobility issues, or live in different geographical locations. Several authors argue that these factors call for changes in how we view nursing home residents’ social interactions. One of the first studies to focus on this issue revealed that organizing video-conference sessions with families increased the quality of interactions – unlike reliance on audio sessions alone – because they made social presence more meaningful (Mickus and Luz, 2002). Remote social contact via videoconferencing tools allows participants to have a better assessment of how others are doing (Hensel et al., 2007). Videoconferencing use with relatives has a positive impact in terms of social support and reducing feelings of loneliness and depression (Tsai et al., 2010; Tsai and Tsai, 2011), thus making residents’ daily life more fulfilling (Tsai and Tsai, 2010). Remote social contact via videoconferencing is considered to be the “second best option for visitation” (Tsai et al., 2010) for residents whose families live in different geographical locations.

Other studies, published during the visitation restrictions associated with the pandemic, have directly or indirectly focused on the remote social contacts of nursing home residents. All these studies have been based primarily on residents’ real or supposed socialization needs (Lemaire et al., 2022). The research questions, often formulated from a negative angle, have focused on the absence of social contact associated with the pandemic context (Ayalon et al., 2020; Eghtesadi, 2020; Gallo Marin et al., 2020; Office et al., 2020; Pachana et al., 2020; Sano et al., 2020; Tsai et al., 2020; Bethell et al., 2021; Bolcato et al., 2021; Gorenko et al., 2021; MacLeod et al., 2021). While it is generally assumed that residents require digital technology to ensure social interaction, these residents’ opinions are never sought. Their views regarding the people with whom they wish to communicate are never clearly explained, despite the fact that the available scientific literature focuses on multiple remote social contacts in a pandemic context (Lemaire et al., 2022). Indeed, several social interventions have been implemented, such as phone calls to older adults by student volunteers (Office et al., 2020), or video-conference sessions by certain associations (Burke, 2020). As part of an action research strategy aimed at setting up quiz sessions via Skype, remote social contacts between residents of several nursing homes were tested (Zamir et al., 2020), and several professionals asked to maintain remote contact with the residents (Pachana et al., 2020; Sano et al., 2020; MacLeod et al., 2021). While several authors suggest that it is important for older people to maintain meaningful relationships (Burke, 2020), or that some residents enjoy receiving calls (Office et al., 2020), it is not clear whether these residents had initially sought these social connections.

Several researchers have identified limitations of digital technologies, such as an inhibited use of videoconferencing due to residents’ cognitive impairment, their physical fragility, and/or their visual or auditory disorders (Ayalon et al., 2020; Moyle et al., 2020; Sano et al., 2020; MacLeod et al., 2021). The digital divide has thus been addressed from several angles, including older adult’s lack of digital skills (Lebrasseur et al., 2021). This divide also concerns the relatives of certain residents (Burke, 2020), especially because social inequalities limit access to digital technologies (Bolcato et al., 2021). Exclusion from technological progress is associated with negative representations of older adults, such as their passivity or their inability to learn (Eghtesadi, 2020). In the case of Telehealth consultations, remote contacts are declared necessary, irrespective of whether or not the older adults show an interest (Marsh et al., 2020). However, having access to technology does not mean that this technology is used in the most efficient way possible (Luscombe et al., 2021). We believe that remote social contact can only have a positive impact on social isolation and loneliness in a pandemic context if professionals support the use of digital technologies or convince the older adults under their care of such technologies’ merits (Sacco et al., 2020).

As several authors have highlighted, this calls for changes in both the professionals’ practices and in the infrastructure, an issue that received little attention before the pandemic (Schuster and Hunter, 2019). Indeed, several limitations have been highlighted, such as professionals being unavailable to set up videoconferencing, their lack of access to such technologies, and their lack of the necessary programming and facilitation skills (Freidus et al., 2020). Staff commitment and turnover is an additional limitation (Gorenko et al., 2021). Several factors must therefore be considered, for instance the purchase of dedicated equipment and infrastructure (e.g., setting up WiFi), the allocation of professional time to help older adults, and the consideration of staffing needs or volunteer training (Bethell et al., 2021).

Digital solutions are thus generally perceived as a more or less effective alternative to face-to-face interactions, albeit by default. They seem to respond to an “emergency” that may be real and/or symbolic, one which is focused on the risk of isolation and loneliness among the older adults, and therefore on the possible deleterious consequences in terms of their health and quality of life. Drawing on a normative (which determines the gap between *what is* and *what should be*) or even ideal (which determines the gap between the means available and the valued and/or valorized expectations) conception of the uses of digital technologies for older adults, there seems to be a tendency for technological solutionism (Morozov, 2013). This conceals the social configurations suitable for significant contact, the unconscious mechanisms at play in these virtual interactions, and the specificities of the institutional context in which they unfold.

### 2.2. Representations of social contact among the older adults, considered from the perspective of the specificities of the nursing home context

Where the older adults are concerned, the risk of isolation and loneliness is an “evil” against which one must fight by making it possible to reflect on the individual and collective representations of geriatric institutions in our societies, which are often negative and marked by loss and death. The feeling of loneliness raises questions about how one relates to one’s own self, deals with loss and separation, and occasionally aspires to be part of a whole, either as a couple or as part of a group, thereby filling existing gaps and preventing individuals from experiencing a certain capacity to be alone (despite the fact that this shapes the experience of dependency). This is because loneliness is less about the absence around oneself and more about the impossibility of addressing this emptiness within oneself. These feelings can be reactivated in cases of isolation, characterized by an absence of social contact and by fewer and less available substitute options when one ages. When the nursing home doors close, the absence of loved ones may increase the risk of psychological, cognitive, and/or somatic decline among individuals who are forced to face an additional loss while in a position of great vulnerability. Indeed, from the outset, life in a nursing home implies a radical and often definitive renunciation of one’s previous life at home, a “home” (Milligan, 2016) which possibly served as a container and as a protective envelope (Eiguer, 2004, 2010, 2016; Zielinski, 2015), and which residents now have to reconstitute elsewhere (Ferreira and Zawieja, 2012; Charras and Cérèse, 2017). This separation also implies a rupture in the usual family and social relationships, as well as a withdrawal from social contacts, an experience likely to reactivate several losses (Riedl et al., 2013; Charazac et al., 2016; Racin, 2019). It also awakens several representations associated with the idea of irreversibility, which is itself frighteningly close to representations of death and to representations associated with the strong heteronomy inherent in these living spaces (Enriquez, 1987, 2006; Talpin and Ploton, 2002; Talpin and Minjard, 2021). Indeed, the representations of these institutions are still primarily defectological, meaning that they are centered on defects. They either view residents as the “dependent older adults,” which is in continuity with the perception of the older adults as sick and mad, or they support today’s ideal of “successful aging” (Billé and Martz, 2010). Consequently, nursing homes are viewed as failing in their socialization mission (*mésinscription*) or, as Talpin proposes Talpin (2021), failing as agents of deregistration (*désinscription*) insofar as older adults subjects in these homes, who often previously belonged to specific social groups (professionally, family-wise, etc.), lose their membership of these groups because of how old age and aging are addressed, on the one hand, and because of the frequent emergence or aggravation of cognitive and/or behavioral disorders, on the other. However, as Talpin (*op. cit.*) argues, these facilities also allow their members to regain membership, albeit of a stigmatized group (Goffman, 1961, 1963) as a result of impairments. These facilities have to deal with the “negative”: negative deposited in their foundation (Pinel, 1996), negative of the older adults people cared for (*a fortiori* dependent), and negative of the professionals, irrespective of whether it is theirs alone or is associated with their encountering the older adults people for whom they care. As a result, a nursing home can only fulfill the healthcare mandate with which it was entrusted over the long term by working on itself, by making “detoxification” efforts, and by endlessly reflecting on anything that may attempt to dismiss – notably through denial and splitting – the negative aspects of castration, loss and death, of which the aging body, which is sometimes dependent and sick, bears the stigmata. A “pact on the negative” (Kaës, 2009) thus emerges in defense of an illusion that may lead to psychic damage and that could be exhausting for the subjects. This calls for intensive and continuous work within the institution to recognize the conscious and unconscious forces in the older adults cared for, as well as in their relatives and in the professionals. In nursing homes, the dependency paradigm (Charazac et al., 2016) shapes the relationships between the residents, their relatives, and the professionals and represents part of the “work of the negative” (Green, 1993) that institutions encounter. This dimension must therefore be considered in order to understand how it is renewed through the way in which older adults residents develop subjective feelings of ownership of those digital technologies aimed at maintaining social ties.

## 3. Methodology

### 3.1. Aims and objectives

First, we need to understand how digital tools can be used to provide a “positive” subjective experience for nursing home residents, by considering the variety of their possible uses. In particular, it seems necessary to analyze the uses that are situated in a functionalist or operational perspective – one focused on the functional dimension and on the operations that it is capable of ensuring – and those that are situated in a perspective that takes into account how these tools are actually embraced and the experiences of those that use them. The manner in which they embrace these tools can lead individuals to use them for purposes other than those for which they were initially intended. The challenge is therefore to specify the conditions under which these tools can be used with a view to promoting and developing the social relationships of the older adults residents.

The question of the conditions of use also raises the question of the *system* in which the technical tool is used, a question inseparable from the tool’s operational or material dimension. Indeed, the system represents the structure established to enable access to the relational situation that videoconferencing targets and to the arrangements chosen to propose, host, make intelligible, and support residents’ commitment to relationships via this form of communication.

Our approach differs from the descriptive and experimental studies which focus on the objective qualities that promote or act as an obstacle to the use of these digital technologies by various actors, the positive benefits of which are presumed from the outset. These studies do not make it possible to obtain a dynamic and relational understanding of the situations of use and non-use, which necessarily involves an analysis of the appropriate socio-technical configurations and the subjective experiences involved.

It therefore appears that the different uses of these digital tools shed light, retrospectively, on the theory of care and on the theory of suffering (Roussillon, 2010), which reemerge here. We posit that these theories are driven by the generally negative perceptions of nursing homes in today’s society. Rather than analyze the effectiveness of the digital approach aimed at maintaining social ties, our study focuses on the processes at play in a situation that attempts to sustain relationships, but which is also *mediated* by digital tools, in order to shed light on how residents embrace these tools. Indeed, the technological support (in this case, video-calls using tablets) is not a simple neutral intermediary. It also promotes relationships (Akrich et al., 1988; Latour, 1991, 1999, 2000), with the participants using it not only to communicate but also possibly to negotiate about different issues affecting their relationships (psychological, social, organizational, etc.).

### 3.2. Mediation as an analytical tool

An analysis of the role that technological *mediation* plays in the relational dynamics of individuals thus appears important, meaning the manner in which digital tools allow the networking of human actors (healthcare providers, residents, relatives) who jointly develop (both humans and artifacts) social ties (Akrich, 1993). On a psychological level in particular, analyzing these uses from the angle of “mediation settings” (Brun et al., 2019; Brun, 2020) provides an excellent opportunity to comprehend the processes involved in the use of these technological tools. For instance, this can help to shed light on the adoption or rejection of these technical supports, or on the diversion from their original use, and thereby highlight the digital realities of older adults nursing home residents in the current post-pandemic society. Mediation is situated in a “space between” – between two spaces, between two moments – and only in this space can it make sense. Nursing-home professionals must also set aside a time and a space dedicated to connecting residents and their families. The mediating object drives transition, and the main effect of mediation strategies is that they bring together the conditions that make access to transitional phenomena possible; (Winnicott, 1951) these may be defined as unconscious phenomena which must be understood within the intersubjective contexts in which they take place. Transitionality unfolds within an intermediate zone of experience that supports the transition between two intersubjective states, making it possible to develop an experience in which one is continuously in rupture, i.e., connected but separated. As Kaës points out, “the consistency of the concept of the intermediary is that it expresses this triple function: bridging a maintained rupture, transforming recovery, and symbolization” (*op. cit.* 20).

### 3.3. Research questions

We therefore reflect on the multiple modes of use of digital tools and analyze the role that these play in ensuring that social contact is maintained in a context that associates multiple needs and desires that are yet to be identified. The question, then, is how likely are digital tools to accomplish a mediating function that connects both intrapsychic and intersubjective dimensions? To this end, we will examine the conditions under which older adults nursing home residents, who are sometimes extremely hampered in their physical, cognitive, and psychic abilities, acquire subjective feelings of ownership thanks to their use of digital tools within an approach that seeks to promote mutual relationships.

### 3.4. Recruitment and data collection

The different configurations of digital mediations seeking to maintain social ties in nursing homes and examined in this study draw on data obtained within the framework of the Innovehpad study undertaken between April and October 2021. This was an interdisciplinary study of the psychological, social, managerial, health, and skill factors that influence the use of digital tools in maintaining social contact among nursing home residents. It was set up following the Covid crisis and the resulting changes in the organizational structure of these homes.

To consider fully the complexity involved in understanding the issues, conditions, and effects of the uses (or non-uses) of these digital tools, each member of the Innovehpad team (composed of researchers in clinical psychology, sociology, management sciences, ethology, and education) drew on their disciplinary knowledge to highlight a specific facet.

In this exploratory study, cases were selected if they corresponded to two requirements: representativeness and comparative reasoning. By “comparative,” we mean the ability to *compare* one situation with another in order to highlight the possible specificities and invariants of “mediation work,” based on singular cases that may later be grouped together into different observations and/or categories. Therefore, our comparative approach does not attempt to apply the same measurement across cases as this implies that the conditions of observation are always equal. It is less interested in replicating observations from a single case to reach a common understanding than in shedding light on the reality and *modes of existence* (Latour, 2012) of the digital tools that seek to maintain social ties in nursing homes. Depending on the levels of integration, comparing cases will then make it possible to identify clusters of the different experiences that give rise to the forms of use (or non-use) observed.

This article focuses specifically on how the association of clinical psychology, sociology, and management science disciplines makes it possible to describe the different configurations of digital mediation aimed at promoting social ties in nursing homes. It then opens the debate on how the psychological issues observed at individual, group, and institutional level may be understood. We visited seven nursing homes in France (including one dedicated to the educational sciences approach) and undertook 210 h of observation and 83 h of interviews. In the 89 interviews conducted, we met with 26 management head of nursing homes, 12 residents, 13 relatives, 36 professionals and 2 groups of professionals (*cf.* summary table of data collection in Supplementary Table 1 and interview guides in Supplementary Tables 2–4). The approaches in clinical psychology and sociology focused on nine mediation situations. Semi-structured interviews were conducted with residents whenever possible, and also with professionals and/or residents’ relatives. The clinical psychology approach relied on semi-structured interviews aimed at assessing the unconscious representations and the psychological or emotional issues around the mediation proposed by these digital tools. The management science approach relied on interviews with nursing home staff (management, executives, professionals in the field) and, whenever possible, with residents and their families.

### 3.5. Data analysis: a relational research method in an interdisciplinary context

These three disciplines sought not only to give a voice to the *actors* involved (residents, relatives, and professionals), but also to describe the *actants* likely to emerge, on whose behalf we would speak. “Actants,” each with a “unique signature” (Latour, 1991, p. 118), refers to the “forms of existence that are involved in a course of action, […] to anything that modifies a given situation by introducing a difference into it” (Latour, 2006, p. 103). While *actors* can speak for themselves, Latour suggests that *actants* do not speak in their own voice: “Because they are mute; because they have been silenced; because, too noisy, they would become inaudible were they to all speak” (Latour, 1984, p. 245).

In this perspective, our work sought to make *visible* unconscious, social, and organizational phenomena by interpreting their characteristics, their modes of expression, and the conditions under which they are used or hindered by human behavior. To this end, we relied on case studies in which each researcher worked alongside the other researchers in the team to interpret data. Our epistemological approach, therefore, makes no attempt to be complementary (Devereux, 1972; Missonnier, 2016) or to highlight a multiple interpretation of the same object. Rather, it is closer to an “extended translation model,” in line with the “sociology of translation” studies undertaken by Akrich et al. (2006). In this model, researchers adopt the position of a *mediator* of an object’s reality, capable of speaking for the *actants*, giving them a voice in places where they would possibly have been voiceless without their help, without their skills and epistemological approach, without this “actor endowed with the capacity to translate what they transport, to redefine it, redeploy it, and also to betray” (Latour, 1991, p. 111).

Our team’s conception of interdisciplinarity leads us to consider, and report on, “mediation” as a “hairy object,” in the words of Latour, meaning an object with numerous attachments, associations, and affiliations. Ultimately, we seek to shift away from descriptions of the substances and properties of this phenomenon to consider “mediation” as a network. In this model, which is inspired by relational ontology (Slife, 2004), actors and objects exist only in relation to each other.

In no way does this choice aim to equate the concept of “mediation” to an axiomatic scheme, generalizing the multiple ways of understanding how the digital tools that seek to maintain older adults nursing home residents’ social ties are experienced. Rather, it seeks to draw on what we believe to be its major theoretical contribution because of at least two essential qualities. First, we believe that it exceeds in promoting and analyzing the *dynamic perspective* in which we would like to incorporate the issues associated with digital tools aimed at promoting social relationships in nursing homes. Second, from a *transversal and relational perspective* – given that the concept of mediation tends both to challenge and to be challenged by different disciplines, notably psychology, sociology, and management sciences – it appears to be a “dialog magnet” between these disciplines, conducive to a reflective and collective production around dynamic issues, particularly individual, group, and institutional issues associated with the digital experience.

## 4. Results: an analysis of the different mediation configurations of the digital tools that sought to maintain social ties in nursing homes during the pandemic period

The results of the Innovehpad study show that the assistance provided to an older adult in a situation of dependency and separated from their relatives can only be “usable” if it meets certain conditions. On the one hand, the tools used must correspond sufficiently to the needs and desires of the individual, which means that they must be assessed before their introduction (based on individuals’ expectations and on what they see as the tools’ benefits) and afterwards (based on individuals’ ability to embrace and use them to satisfy the needs or desires unexpressed until then). On the other hand, the approaches proposed must help individuals to embrace the tools proposed, and this undoubtedly requires some psychological work. Such an approach can make it possible to reduce the gap between residents’ experience of the tools and their acquisition of subjective feelings of ownership of these tools. Lastly, given that these tools cannot be effective in themselves, the system must be able to rely on specific material and organizational arrangements. Special attention from the institution is thus required.

It is under these conditions, which are intricately linked, that one can examine the digital mediation approaches that seek to maintain social contact and to ensure that the older subject has a positive subjective experience, irrespective of whether this outcome is expressed by the subjects themselves, by their relatives, or by professionals. Our results show that the situations of “failure” – which often lead to abandonment – and the situations of “success” – which lead to regular or occasional use – cannot be attributed to weaknesses evaluated independently, whether physical, cognitive, psychological, or social weaknesses. To succeed in the mediating role, i.e., in mediating a sufficiently good relationship between residents and their loved ones, there is a need for more than merely efficient and ergonomic digital tools, and for more than just cognitive, sensory (particularly sight and hearing), and physical (visual-manual coordination) capacities: it requires specific organizational, interactional and psychological configurations.

### 4.1. Mediation configurations according to the organizational context of nursing homes

The attention paid to the different categories mentioned above calls for the identification of the different types of mediation possible based on the organizational context of nursing homes. Indeed, the organizational culture, the nature of the leadership, and the history of the organization all play a role in the development and evolution of organizations. This article analyzes the mediation proposed between residents and their relatives, which is provided by professionals and backed by the nursing home management. We identified three types of mediation which, while neither exclusive nor exhaustive, can be understood as ideal types.

#### 4.1.1. “Fake mediation”: professionals simulate ties with relatives

Nursing homes were put in the spotlight during the hyper-mediatization associated with the Covid-19 pandemic. Some of these homes experimented with mediation of the ties between residents and their loved ones in an attempt to manage risks. They proposed a stage setting showing what should be seen, with actors in front of screens and dialogs that were occasionally overheard, aimed at reassuring loved ones rather than at nurturing the ties of residents who had requested remote contact with their loved ones:

Often, the tablets were more for the people, for families than for the residents here. They were supposed to reassure them, so that the relatives could say to themselves: “Well, they are not telling us lies, she’s fine.” I feel like this was the main function at the start. (Interview Nursing Home 6: Researcher in management sciences – C35, Woman, 50–55 years old, management head, living in rural area)

It was good for the family, but not for the resident. Well, to reassure the family, but not the resident… He did not understand. At least not in the Alzheimer’s unit. (Interview Nursing Home 6: Researcher in management sciences – C38, Woman, 25–30 years old, logistic officer, living in rural area)

In this context, while residents did not necessarily seek digital technologies, they could use them to facilitate the relationship with professionals, or to please or obey them. The question of freedom of choice thus arose:

Resident’s daughter: Relationships on tablets? But she does not want it.

Interviewer: And yet she accepted them during the confinement period?

Resident’s daughter: Well, uh, yeah… Because she wasn’t the one holding the tablet, it was the facilitator who was with her and who handled everything.

(Interview Nursing Home 2: Psychology researcher – B14, Man, 60–65 years old, retired, former sports teacher, living in peri-urban area)

At the time, the managers of these establishments were concerned with legal or reputational issues: they sought to avoid complaints, to look good, to keep proof of interactions in case of incidences of geriatric cachexia, and to protect themselves from criticism. This type of technological mediation was therefore somewhat deceptive. It took place in environments in which only a few professionals were available to help the residents, with time constraints making it secondary to healthcare:

We do not have the staff to really…let us call it, build ties. So it’s true that there are many healthcare professionals who are a little frustrated and who tell me “but we only do… that’s the only thing we do, it’s non-stop work. (Interview Nursing Home 6: Researcher in management sciences – C36, Man, 35–40 years old, nurse coordinator, living in rural area)

#### 4.1.2. “Humble attempt at mediation”: professionals become the contact

Some nursing homes revolved around an “activist” or “humanist” approach and developed specific resources to establish projects that made it easier to listen to residents’ needs and demands. Professionals were thus expected to take the necessary time to ensure the holistic support of residents. Irrespective of whether the objective was to rediscover a “family spirit” (nursing home n°6) or to adopt a gerontological approach inspired by the model of institutional psychotherapy (nursing home n°2), the heads of these homes set the stage for personalized, in-depth, and meticulous support which professionals provided.

One nursing home management head explained that digital mediation began when a family made an appointment, after which the resident was notified. He stated that:

The professionals prepare, they warn the person, the resident, saying, for example: ‘Mrs X, you have a Skype call in 15 min; are you still OK with that?’ because, you know, sometimes there are people who are indisposed, who are... who aren’t feeling well, so we try to assess the potential success of the encounter. (Interview Nursing Home 4: Researcher in management sciences – C33, Man, 35–40 years old, management head, living in urban area)

The facilitator then established contact and helped the residents who were unable to manage the video-call by themselves:

One has to stay close by, explain to them what’s going on: “Look, there’s your daughter on the tablet, you can talk to her”; or try, for example when the resident does not hear very well, he’ll say, she’s asking you this, she’s showing you a picture. (Interview Nursing Home 4: Researcher in management sciences – C33, Man, 35–40 years old, management head, living in urban area)

The mediation provided by professionals via digital technologies could then lead to genuine contact, to a close relationship between residents and their loved ones. Many professionals reported the visible satisfaction of residents during remote interactions:

Many residents were happy to see their family via Skype. (Interview Nursing Home 1: Sociology researcher – A8, Woman, 80–85 years old, resident, living in peri-urban nursing home)

When some residents see the faces of their daughters or their sons, everything changes immediately. The eyes light up, they are happy. Uh… Their joy is immediately visible. (Interview Nursing Home 1: Sociology researcher – A16, Woman, 45–50 years old, social life assistant 1, living in peri-urban area)

It was not always possible, or desirable, to put people into contact, and this was a fact that some professionals deplored, occasionally having to put an end to videoconferencing sessions:

For me, the objective is to maintain social contact, but if for 45 min you have the resident who remains stuck on their story about breakfast [unsatisfactory], and on the other side the girl who is completely desperate and who cannot talk to her mom, and the facilitator who’s trying to guide the discussion, who’s trying to explain videoconferencing, look, there’s your daughter, you can talk to her live, she’ll answer you, etc., well, it’s true that sometimes, for some, there could be... well, we do not achieve the objectives, in any case the objectives or the objective of social contact. (Interview Nursing Home 4: Researcher in management sciences – C33, Man, 35–40 years old, management head, living in urban area)

The professionals thus adapted to the situation to ensure that residents were comfortable.

In some cases, they were sufficiently independent to manage communication without the help of professionals, as one resident reported:

We would see the images, first of the girls [the nursing assistants] and we would talk. The girls left and we were able to talk together [with her children]. [....] When you have Skype, you can talk about whatever you want because the girls leave and close the door. (Interview Nursing Home 1: Sociology researcher – A8, Woman, 80–85 years old, resident, living in peri-urban nursing home)

In other cases, the professionals had to stay, but this occasionally led to discomfort:

Sometimes it bothers me to be there, because I tell myself that, because I’m around, they may not dare to talk about certain things. And at the same time, there is not much choice. Never has a family reproached me for staying… But it’s me, it’s how I feel. It’s their moment, but there’s still someone around. (Nursing Home 6: Researcher in management sciences – C37, Woman, 45–50 years old, social coordinator, living in rural area)

The tact necessary to ensure the success of this mediation was clearly expressed by one facilitator who spoke of the embarrassment that the present/absent place could cause when connecting residents with their loved ones.

In these contexts, the contact established via digital tools is nourishing for the residents. Although some of them fail, attempts to meet the residents’ demands for contact are made by professionals. This approach, which revolves around residents’ needs, requires the management to make changes in the organizational structure, notably with regard to an adequate pace of work and effective management which promotes professionals’ work while respecting the older adults that they care for.

#### 4.1.3. “Substitutive mediation”: professionals as the link

Irrespective of the organizational structure, certain residents viewed the use of digital tools as too remote, too strange, or too disturbing:

For them the internet is science fiction. (Interview Nursing Home 4: Researcher in management sciences – C34, Man, 35–40 years old, management head, living in urban area)

While the connection observed was real, important, and nourishing for the residents, it concerned residents and professionals, rather than residents and their loved ones. One facilitator spoke of the case of a resident who began speaking with her, rather than with her loved one who was present during a video call:

She admires this technology […] every time she gets into a debate about it. She says: “Ah, it’s a good system. And can we do this with everyone?” So she would stop listening to her daughter. (Interview Nursing Home 6: Researcher in management sciences – C37, Woman, 45–50 years old, social coordinator, living in rural area)

One psychologist also shared the case of a resident living in an Alzheimer’s secure unit after contracting the disease relatively early. This resident used to walk with her daughter, sometimes in silence, leading to the establishment of a strong bond. However, this contact became impossible after the visitation restrictions. The psychologist established remote contact and then walked with the resident while holding the tablet in her hand. An important connection was thereby established with this professional.

This example was an important reminder that residents’ social ties are embodied in their relationships with professionals, notably those professionals in the logistics department responsible for cleaning. During our observations, we noticed that these cleaners knew the residents, their habits, and their tastes quite well. During an immersion session, one of these cleaners accompanied us to a resident’s room where the television was on. She then exclaimed:

“But, Mrs. XX, are you watching TV? This is not the time that you normally watch. Do you want me to switch it off?.” The resident, who was not listening to the program, agreed. This type of interaction involved a resident and a professional and was transposed, as mentioned above, in the case of digital mediations

In other words, when professionals simulated a link between residents and their relatives, digital mediations were “fake”; when these mediations made a genuine attempt to *establish contact,* a nurturing relationship could arise, and when *they were the contact*, the mediation was substitutive: contact was established between the residents and the professionals, rather than between the residents and their loved ones. These types of mediation relied on the existing organizational structure; indeed, the introduction of digital tools was not disruptive for organizations. On the contrary, it made it possible to reveal previously existing mechanisms.

### 4.2. Mediation configurations according to the interpersonal interaction approach

We observed several types of socio-technical mediations made possible when residents and their relatives were connected, with the professionals acting as intermediaries (Battentier and Kuipers, 2020) who guided the process while participating in the production of remote relationships.

#### 4.2.1. The “one-way window”

In several situations, the mediation produced could be perceived as a “one-way window,” meaning that, above all, it allowed relatives to reassure themselves as to the physical and mental state of residents, without the latter actually participating in the exchange. In this sense, for instance, a psychomotrician explained to us during an interview that:

For the girl, it was really important to see her father. In fact, it was… I think it was really good, it’s just that it was… Well, I was a little surprised at how hard he found it to embrace… to embrace this tool, whereas it seems super simple to me who is a millennial. I saw that it wasn’t easy for him. [...] Actually maybe it was easier for him on the phone because he was more used to it, and when we switched to the tablet he was a little taken aback, he did not really know what to do. [But] it was better for his daughter, seeing him reassured her. (Exploratory interview: Sociology researcher – A4, Woman, 30–35 years old, psychomotor therapist, living in Paris)

#### 4.2.2. The “counter window”

Ideally, the mediation proposed may be perceived as a “counter window,” meaning that the residents and their loved ones were able to see and talk to each other without risking contamination. For example, during the interview, and following our request, one resident was quoted as follows:

Interviewer: What I would like to know is what you talk about during video calls, does one experience the same thing as they do when they see each other?

Resident: Yes, yes, for me, yes.

Interviewer: You told me, for example, that when your daughter came, you would talk about the news. Do you do that too?

Resident: Yes, we would, yes. We would talk about the news of the week. [Especially in relation to the Covid] since that was the main topic.

(Interview Nursing Home 1: Sociology researcher – A9, Woman, 80–85 years old, resident 1, living in peri-urban nursing home)

In such a situation, professionals were essentially responsible for launching the videoconference call. They would then withdraw to promote the emergence of intra-family intimacy. However, the remote social contact thus produced was described as a degraded version of what real-life visits allowed. Indeed, the same resident said: “No, I prefer face-to-face however.” She specified: “Because we see the person, it’s much better.” Even though videoconferencing makes it possible to “see,” her discourse implied that this type of communication was a degraded version of the possibility of “seeing,” that the physical body was missed.

#### 4.2.3. From the “virtual smoke screen” to the “opaque counter window”

Depending on the different actors involved, videoconferences were perceived as “virtual smokescreens,” as with the case of a resident and her son who were both frustrated with digital technology. She said:

I cannot get used to it, [...] because it irks me (Interview Nursing Home 2: Psychology researcher – B9, Woman, 90–95 years old, resident, living in peri-urban nursing home).

After two attempts, the son, a retired teacher who had fought against the use of digital tools by his students, told us that their telephone discussions were more than enough to allow him to “see [his] mother every day” (Interview Nursing Home 2: Sociology researcher – A32, Man, 60–65 years old, resident’s son, living in peri-urban area, retired-former sports teacher). The mediation of the relationship via the telephone could then be considered as an “opaque counter window” which allows one to talk without an image interfering with the relationship. The son’s slip of the tongue, when he specified that he was able to “see” his mother, is therefore significant. For this same resident, however, the mediation of the social relationship with her great-grandson via videoconferencing appeared to be a “counter window” in the sense that it was the only way to communicate with him as he was too young for a telephone call. The relationship therefore primarily involved gestures, with the child pointing to the resident and the latter waving.

### 4.3. Mediation configurations depending on the quality of the transitional space

Focusing on the unconscious dispositions mobilized and supported via digital tools also allows us to go beyond material, organizational, and interactional arrangements and to examine the intrapsychic and intersubjective processes that help individuals to embrace these tools. This is only possible if the tools are proposed and used within a transitional space developed via digital mediation. But, first, the environment must be made as accessible and as convenient as possible for the older dependent person and must allow transitions. The responses from the environment are thus the vectors of individuals’ subjective experiences revealed during the mediation situation, and also powerful drivers of their transformation. When these responses offer an objectual support sustaining the construction of a potential transitional space, the device possibly becomes a shared co-creation between each actor involved, between the real and the imaginary, between the internal and the external worlds. Otherwise, it can be experienced as an attack on or imposition of objectively perceived external reality, or as a “subjective object” retained in the subject’s sphere of omnipotence and acting as a set of projections disengaged from their perceptual roots.

#### 4.3.1. “Playing the game”

The pleasure felt, expressed, or revealed in either speech or behavior is a valuable indicator of individuals’ ability to function within this transitional space and of the quality of commitment to the technology, even in cases where they did not initially request the tool. Some people thus agree to “play the game,” their desire revolving around that of their loved ones and/or the professionals. They discover a mutual interest in these situations, or even a shared pleasure in a sufficiently good relationship between residents, relatives, and professionals. The absence of pleasure is a common feature in the configurations described earlier, which function as “smokescreens of the virtual.” Digital tools can only act as mediators if they are sufficiently *emotionally* invested in by residents, their families, and the professionals, and if their internal organization and the unconscious dispositions of the different actors make the connection between intrapsychic and intersubjective networks possible:

A nurse thus spoke about how her rejection of digital technologies in her own daily personal life, and her feelings of incompetence, may have prevented her from committing to tools that she nonetheless perceived as being useful and important in enabling residents to maintain contact with their loved ones. She also said this may have led her to develop avoidance behaviors so as to avoid these digital tools. Beyond the obstacle of digital skills, the clinical interview revealed additional obstacles, relating this time to the feelings of rupture that the implementation of videoconferencing provoked among residents in need of individual support during this time. This rupture was in continuity with the care provided in the nursing home (mediation obliged professionals to leave the service and to join a space dedicated to videoconferencing, which the assistant viewed as unfair to all those who needed her during this moment, i.e., other residents and her colleagues). It also affected her previous relationship with this resident’s family. She specified that she usually met many families by acting as a mediator for residents, meaning that she had discussions with visitors within the common spaces of the nursing home, or even in the corridors. There were often other residents and other families participating in discussions in the space where this professional was assigned: in the space–time continuum of the video-conference, in a special meeting with this resident’s family, one filled with renewed intimacy, irrespective of whether she was called upon or whether she was merely a witness, a spectator to the discussions. This nurse voiced her discomfort in this paradoxical situation in which she felt like an included/excluded third party, and which tempted her to pry. The new structure no longer allowed the resident-relative-professional relationship to encounter the organizers who had previously provided a framework for a sufficiently good triangulation, and who particularly relied on the mediation provided by the institutional context and the presence of other residents, visitors, and professionals. (Interview Nursing Home 1: Psychology researcher – B7, Woman, 45–50 years old, nurse, living in peri-urban area)

This testimony clearly illustrates how digital technologies are based on both material and unconscious dispositions, the highlighting of the inaccessibility of some dispositions sometimes concealing the inaccessibility of others. Although insufficient, making an emotional commitment to video calls is essential for the success of this intermediate relationship resulting from the physical distance between, and the separation of, the interlocutors. Digital technologies can only act as mediators if they meet the conditions enabling the creation of an intermediate space capable of restoring the transitions between separate spaces (residents’ living spaces / relatives’ homes): if they make it possible to create continuity and provide the same quality as the previous relationship between residents and relatives and between relatives and professionals; and if they support the connection between residents’ unconscious experiences and their external worlds, between the processes of projection and those of perception, called on to interpret the image and sound generated by videoconferencing.

In certain mediation frameworks, digital tools allow the creation of an additional real space which supports the psychological process of “presentification” (Haddouk and Missonnier, 2020), i.e., which makes it possible to re-establish a connection between one’s awareness of unconscious processes and what one feels in one’s body. This work is particularly undermined in neurodegenerative disorders, which explains why the professionals undertook this work only with residents suffering from moderate to severe cognitive disorders. Indeed, some professionals remained physically present during discussions between residents and relatives, under the pretext that this would enable them to be immediately available should a technical problem occur, and thus they positioned themselves as assistants. Others took on an auxiliary ego function, offering themselves as an “other,” as a *loved one* ready to lend their body, their voice, their time, and their “psychic space” to support the illusion of continuity in this connected but separated long-distance relationship:

This was the case with the “substitutive mediation” mentioned above, in which the professional walked with the resident whose reunion with her daughter had previously revolved around walks both within and outside the nursing home, and also occasionally sang along with her. During the Covid-19 confinement, the professional (psychologist) succeeded in making this digital tool usable – it had otherwise been inaccessible for this resident who had major cognitive disorders – by holding and carrying the tablet herself, moving with it from one place to another, and changing its position depending on the movements and initiatives of both the resident and her daughter. She also reformulated, or repeated, certain words, sounds, or melodies while relying on her observations to relate to the experiences of the resident and her daughter, as well as to the tone of the mother-daughter bond. The professional adjusted her intervention according to the effects of presence or absence that this aroused, in order to help rediscover or create the quality of the previous relationship between the resident and her relative. She focused on a mediation approach which borrowed from, and conveyed, an affective and sensory tone that quickly reinforced the relationship between past and present, and between visual and auditory perceptions and bodily experiences. (Interview Nursing Home 1: Psychology researcher – B3, Woman, 30–35 years old, psychologist, living in peri-urban area)

The professional’s non-encroaching proximity thus ensured some form of continuity in the emotional, affective, and fantasy behavioral divide common between face-to-face and distance relationships (Haddouk and Missonnier, 2020). In addition to relying on her knowledge of the quality of the previous relationship between the resident and her relative, she also relied on a position ultimately less substitutional than that of a third-party, and one that was respectful of the distance required to ensure that the different spaces were maintained; this helped to structure this three-way relationship. This situation reflects the inventiveness and creativity that certain professionals and relatives managed to “find-create” (Winnicott, 1971) every day, often in an intuitive and spontaneous manner (according to the interviews we conducted), in order to give substance to the “presence of the absent,” to allow the person to feel they were in the presence of someone who was actually distant. The frameworks that appeared to most favor residents’ ability to assimilate this experience subjectively were those in which the digital tool – including the positioning adopted by the professional – was designed in such a way as to maintain the illusion of continuity (between oneself and the other, between what is perceived and what is projected by the image appearing on the tablet, etc.) – as well as the consubstantiality of the pleasant and unpleasant aspects of this experience – or to favor access to transitionality and ambivalence.

#### 4.3.2. Disturbing strangeness

A minimal integration of contrasting instinctual drives directed toward the tool appeared essential to reduce the degree of surprise, the strangeness, and the anxiety occasionally aroused by the tool’s effects, against a background of permanent change. Looking into the screen’s mirror and discovering an image of someone other than oneself, thus breaking the illusion of completeness and limitlessness, may have led to wild interpretations from certain residents. This may have aroused a disturbing and intense strangeness around “that, within us, which must be set aside, rejected, disavowed or left unspoken, but is suddenly revived, not without displeasure” (Green, 2000, p. 177):

For instance, one resident told us how, positioned in front of her reflection on the tablet’s screen, she not only recognized herself as she was, but also had to face the perception of a body that irreducibly escapes attempts to master work around one’s own death; in front of her reflection, she had to face an open breach in the manifestation of the temporal uncertainty of one’s being.

This mirror effect, which tore off the protective veil of the fantasy, awakened a castration anxiety so intense that repression was insufficient to contain the excitement and she was forced to remove herself from the perceptive field. This led her to withdraw from the digital project and subsequently to reject it. There seemed to have been no discussion between professional and resident to prepare the latter for this potential outcome and to co-construct some form of background allowing perception-projection processes to be played out in conditions offering subjective security, thus safeguarding residents’ narcissistic continuity. (Interview Nursing Home 1: Psychology researcher – B2, Woman, 80–85 years old, resident, living in peri-urban nursing home).

Here, as well, the quality of the mediation provided by the professional played an important role in determining whether this experience took place in a context where doubts were sufficiently addressed. In this respect, most residents expressed the extent to which this experience could arouse contrasting emotions, particularly joy and sadness, especially when it was time to separate. Once again, the manner in which this separation took place depended on how professionals paid attention to this specific moment:

For instance, the interviews undertaken with a facilitator highlighted how difficult it was to bear and embrace the “normal” sadness associated with this separation without excessive sentimentality or the triggering of manic defenses, which was behind the ideology of the “heroic professional” at the center of political and media attention. (Interview Nursing Home 1: Psychology researcher – B12, Woman, 45–50 years old, social coordinator, living in peri-urban area). Other professionals spoke of their encounter with the pain, even the distress, of certain residents in certain situations. These were when the screen became empty after the image of their relative disappeared when the tablet was switched off, when a loved one was sometimes barely recognized – especially when residents had cognitive disorders, for instance during early stages of dementia – or when they were recognized in a way that made residents feel they were experiencing a disturbing strangeness. (Interview Nursing Home 1: Psychology researcher – B3, Woman, 30–35 years old, psychologist, living in peri-urban area / Interview Nursing Home 1: Psychology researcher – B6, Woman, 30–35 years old, reception officer, living in peri-urban area)

#### 4.3.3. Excess contact

Providing a facilitating environment, and allowing digital tools to provide a significant subjective experience for residents and relatives, requires one to address the ever-present temptation of excessive seduction or compassion and reassuring complicity. One must also avoid the occasional infantilization of the resident, which prevents use of the forcefulness required in the face of a substitute relationship while awaiting the lifting of visitation restrictions, a situation that is inevitably frustrating, disappointing, and unsatisfactory, in part at least. A facilitating environment also requires one to avoid overinvesting in gratitude (particularly expected from relatives) as recognition of a job well done, or of proven usefulness (see the “fake mediation” configuration proposed above), at the risk that recognition – which is essentially about *doing* (doing with, doing for) – becomes more operational, and that the psychoaffective stakes of the tie are denied. In some configurations, the tie appeared as inherent to the relationship rather than as an element that must ceaselessly be reactivated in the face of change; the digital tool was then proposed (sometimes imposed) without working on the connection first. This assumes a minimal investment and a necessary consent or assent (des Lefebvre Noettes, 2018) from the residents. This concern is not only an ethical *praxis*, but also requires a clinical appreciation of the motivations and effects of this commitment. We thus focused on how the implementation of videoconferencing tools has occasionally relied on excessive anxiety, where seeking the good of others has led to a focus on incapacities and to the imposition of several constraints (even relational) in the name of “care.” Certain residents’ attitudes of refusal, submission, or disinterest show the extent to which seeking the good of the other is not sufficient for the other to accept consideration toward them, to find it meaningful, or to express themselves, even via a refusal not overly tainted by guilt:

For instance, a resident expressed her annoyance at what she perceived as an “order to connect” via videoconferencing, which she viewed as “excessive contact” compared with her previous relationships with her relatives. This excessiveness prevented the implementation and adoption of a serenely consented passivity which would have allowed her to develop a bordered and fertile hollow in which to take refuge, in support of *suspension* rather than *inertia*, and aspiring to the elimination of agitation, torment, and suffering. (Interview Nursing Home 2: Psychology researcher – B10, Woman, 90–95 years old, resident, living in peri-urban nursing home)

Some residents appeared to remain as they always had been throughout the duration of confinement, as though protected from the surrounding anxiety-provoking environment and the conflicting issues of the common solicitations that habitually increased – particularly in nursing homes – an ideal of autonomy in terms of activity and movement, and which were now turned toward protection, with the confined spaces of their bedrooms perceived as the safest spaces.

The systematic use of video calls in certain nursing homes – irrespective of whether or not these tools were aimed at acting as mediators to help residents to maintain their relationships – appeared to be an attempt to offer loved ones an object that was always accessible and was quick to hear, to respond, to reassure (sometimes by showing reassurance), or to relay, in a massive counter-cathexis of absence commensurate with major separation anxieties and their deadly echoes, particularly in geriatric facilities. In many respects, the proposal of “contact at all costs,” occasionally overinvested in by professionals, seemed to be in response to the perceptions, sometimes unstated, of renewed castration anxiety, passivity, and even death among residents, their relatives, and also themselves. In this context, the interviews highlighted the extent to which some professionals found it difficult to respect residents’ defensive behaviors, some of which involved withdrawing investment, opposition, and refusal. In these situations, some professionals may have had difficulty maintaining the quality of a relationship that was not excessively demanding. Some also had difficulty in sustaining an investment that was experienced less in the quantity of presence or in “doing” than in the realization of a presence/absence background that tested the question of the *reliability* of the objects to be invested. These individual, group, and institutional difficulties draw attention to the psychological, relational, and organizational conditions required to ensure that the mediating potential of digital tools, aimed at maintaining social ties in nursing homes, does not disappear through the effects of subjectively perceiving activism as an infringement that does not allow individuals to give meaning to what is experienced, acted on, felt, desired, or refused.

## 5. Discussion: from inequalities in the adoption of digital tools aimed at maintaining social ties in nursing homes to misunderstood demands

### 5.1. Digital technology: a “non-neutral” intermediary

The Innovehpad research built on studies that emphasize the need for a shift from analyzing the relevance of technological tools to an analysis of how these tools are proposed and used (Wu et al., 2011; Sharkey and Sharkey, 2012). Several studies, such as those undertaken in the philosophy of technology by P. Verbeek (2005, 2009), have attempted to highlight the social and cultural role of technology, as well as the ethical and anthropological aspects of the relationship between human beings and technology. A similar question, which clinical psychologists have addressed from the angle of the “non-human environment,” has aroused less enthusiasm in this community, even though previous studies by H. Searles (1960) attempted to analyze how this environment affected the unconscious. However, as Missonnier argues, “scotomization is significant because not only are the representations supported by the common technical objects that they produce, but they are simultaneously sculpted in return by their customary relationships with them” (Missonnier, 2009, p. 232). Indeed, these technological tools are much more than silent and passive objects used only as instruments, or as a narcissistic extension of the user’s capacities; they affect individuals from whom they expect a reaction. While the Covid-19 pandemic has forced many countries to pay attention to e-health and to remote devices, it has also made it possible to increase the call for studies in the clinical psychology and psychopathology of digital relationships, a field in which few or no studies have been undertaken. Clinical psychologists working in this field have essentially focused on analyzing the psychic processes associated with certain therapeutic tools such as digital mediations via video games (Gillet, 2018; Haza, 2019; Gillet and Jung, 2022; Haza and Hung, 2022; Tisseron and Tordo, 2022). Others studies have analyzed the uses of digital technology, undertaken a psycho(patho)logy of our daily virtual reality (Tisseron, 2012; Vlachopoulou and Missonnier, 2019). More recent studies reflected on the potential and the particularities of digital psychological assessment (Bravermann and Vlachopoulou, 2023).

However, the manner in which older subjects relate to digital technology has received little interest from clinical psychology, despite the fact that this would allow a better understanding of the specific unconscious mechanisms mobilized when digital tools are proposed to older adults, their relatives, and professionals in nursing homes. While there have been passionate debates around these questions, there is still no clear distinction between technophiles and technophobes.

The findings of our study are consistent with recent studies pointing out that, while digital tools transform professional practices, they also lead to some form of ambivalence associated in particular with the introduction of digital tools in the relationship of care and in nursing homes (Gaglio, 2018; Dussuet et al., 2022). In addition, we found that no professional had received training in the use of these technical tools, through activities focused on the relationship dimension. Professionals used their everyday experiences to set up “experimental” approaches that were based on improvisation and creativity, and they also relied occasionally on inter-professional mutual aid to promote the development of certain skills. This availability was, however, largely restricted by the heavy workload associated with the well-known difficult working conditions in nursing homes, which became even more difficult during the pandemic. The study, however, shows how training must go beyond technical issues and must pay attention to deepening the relational issues that have emerged alongside these new digital technologies. The report on the ethical and legal issues of the use of digital technologies among older people, commissioned by the Silver Economy Sector (Brugère and Gzil, 2019), also highlights the need to go further with regard to certain ethical concepts and certain rules relating to rights and freedoms, notably in relation to changes in the legal framework of professional secrecy and information-sharing.

At a time when the material conditions of care, and the intermediate spaces (Gaillard, 2017) within these institutions, are greatly threatened and at risk of exclusion, technological tools may become symbolic of the expression of a “malaise in care,” provoking tension between the mandate of care and the mandate of protection entrusted to these institutions. Numerous aspects for or against autonomy, experienced in a situation of dependency, are therefore likely to permeate the encounter when videoconferencing is used, and these may or may not provoke conflict, depending on whether nursing homes are willing and able to develop the capacity for containment and transformation. For healthcare providers, this *care* (within which videoconferencing falls) must itself be addressed.

This raises questions about the institution’s *desire* to reduce the constraints weighing on the subjectivity of and crushing professionals’ psychic temporality – making it possible to subvert technological tools – and to situate the different actors and actants as objects capable of acting as mediators rather than as neutral intermediaries. Metabolization, or even psychoanalytic elaboration, can then be undertaken, individually and as a team, to mobilize the individual and collective resources that increase the chances of success. These resources must be able to bring transitionality into play and to integrate contrasting instinctual drives within a conflict of ambivalence which affects, as some of our results show, the investment in these digital tools. The identification of these two drivers, ambivalence and transitionality, is extremely valuable in helping one to acknowledge the experience of separation that inevitably induces discontinuity. However, this is a bearable discontinuity insofar as the illusion of continuity can be maintained within the transitional space, notably by supporting symbolization processes, the quality of which future research should clarify. These drivers allow the constituent objects of the technological tool, recognized in their radical otherness and foreignness, to promote the satisfaction of instincts, but they also become an unending source of frustration. This perspective is in accordance with the function of the *pharmakon* screen (Vlachopoulou and Missonnier, 2019) – remedy and poison – in which the digital object leads to strong ambivalence and is experienced as both useful and dangerous for relationships, as both nourishing and toxic.

### 5.2. The misunderstandings of the videoconferencing request

Under favorable conditions, the material and psychic provisions that the tool combines make it possible to develop an intermediate space of experience, one which opens up a potential transitional space characterized by the fact that it belongs to both the real world and the fantasy world, to both external reality (material and environmental) and internal (psychic) reality. In other words, making use of transitionality notably involves promoting and committing to a space of illusion, a space of all potentialities, in which the question of “who does what,” or “who wants what,” is not directly asked. In this regard, when the residents were questioned directly on their request for videoconferencing, very few expressed – when they remembered engaging in this experience – a commitment to this tool or a “desire to use it.” While several residents struggled to explain the reasons for their refusal or reluctance, others mentioned some form of coercion, the desire coming from a stronger other, on whom one depended. The latter therefore accepted the proposals for fear of retaliation or of being abandoned.

This calls for particular attention to be paid to how residents’ requests (or non-requests) are received, heard, or possibly transformed, in order to identify whether, behind the manifest behaviors and discourse, lies a latent request that may be struggling to express itself. The complexity of this request may be explained by the fact that it lies at the crossroads of needs and desires and is associated with an anxiety that shapes the differentiated satisfaction of instincts, resulting in narcissistic – or objectual – and specific and singular investments in the object. Thus, the manifestation or expression of a resident’s complaint of loneliness (“I feel alone”) may have been interpreted by relatives and/or professionals as the expression of a need or desire for contact. This interpretation, in which the projections from one’s entourage are woven, may have given rise to the proposal to connect with loved ones via videoconferencing.

Such a response, however, can be understood as a defensive mode of addressing guilt and anxiety among relatives and/or professionals, in response to the anxieties of castration, abandonment, or even death among the older adults, which were undoubtedly heightened during the confinement and health threats, but which are already central to the unconscious issues associated with dependency and institutionalization as one ages. When these anxieties are not worked out, the decision to set up videoconferencing, with its underlying defensive objective, reveals some form of “mirror functioning” in which professionals mirror the older subjects with whom they work, in line with the “*functional homology”* (Pinel, 1996) phenomenon. By mirroring the anxiety of the older subject (or that of their loved one), videoconferencing, while seeking to produce a positive effect, risks displacing and increasing the effects of the “negative” of dependency, which may worsen individuals’ issues within nursing homes.

### 5.3. The different configurations aimed at using digital tools to promote social ties: an indicator of how the “work of the negative” of aging and dependence is addressed in nursing homes

We believe that the situations in which older adults nursing home residents rarely participate in decisions about setting up videoconferencing sessions, or in which their voices are rarely heard, reveal how dependency shapes the relationship between the different actors, or more precisely, shapes the myths and representations around addiction that structure the relationships between different actors. Participation in decision-making can take different forms and can range from initiating a request to expressing consent or satisfaction (even in cases where there is still some ambivalence). This issue seems essential for a better description of how these tools can help to promote a “positive” subjective experience for older adults residents. It must be said that this positive valence is closely linked to actors’ representations and aims when they start using this tool and is also connected to the theory of care that underlies professionals’ actions: does this positivity help to promote residents’ well-being, their pleasure, and their autonomy? Does it compensate for their incapacities or help them to express their unconscious mind?

The different ways in which the digital tools aimed at the maintenance of social ties in nursing homes are used thus shed light on the ways in which residents’ requests to engage/be engaged in this proposal are considered. The situations in which the request was not considered, or in which consent was not sought, thus raise the question of the strong heteronomy in these institutions, when dependency and autonomy are opposed back-to-back, which is occasionally hidden behind all the best reasons for providing care. They also raise the question of how the relationship between residents, families, and professionals is structured and incessantly plays out as soon as the older adults joins the nursing home. Digital tools can only act as mediators if the relationships between residents, their relatives, and professionals are organized around a “good enough” structure comprising three parties. There must also be mutual support between the family group and the group of professionals, the latter also depending on the support of the institutional system. This condition requires the adoption of changes by the institution and by the professionals, notably regarding their ability to offer spaces for transformation that allow the “nesting of frameworks” (Kaës, 1987), and of family and institutional frameworks in particular. This support can be compromised by the psychic conflict arising from the “horizontal tension” between the various members of the family and the professional entourage, on the one hand, and the “vertical tension” produced by the relational dynamics between residents and relatives, on the other. As the results show, the objective of videoconferencing can then become counterphobic, i.e., aimed at reassuring feelings of guilt and fantasies of abandonment and mistreatment, but also at proposing some form of control via the visual, or the scopic.

In these configurations, the initial aim of “social contact” seems relatively present in reality compared with what was initially laid down or expected. Our study thus shows the extent to which the feasibility of setting up digital technologies may prevail over the question of the legitimacy of social contacts, thus overlooking the following essential questions. Is social contact desired? Are all residents capable of finding, creating, and committing to social ties? How does each individual define “social contact,” or perhaps a privileged moment spent in the presence of the other whose otherness is not always acknowledged but whose presence awakens emotions and feelings of pleasure, of being alive, of being in a safe space, even if under the constraints associated with Covid-19 confinement or with the digital tool that is viewed as unwanted, unpleasant, complicated, frustrating, or disappointing?

## 6. Conclusion

The results of the Innovehpad research show that videoconferencing tools, while non-invasive in a physical sense, cannot claim to be neutral in a psychological sense: the technologies that attempt to act as mediators in maintaining or developing the possibilities of social contact between older adults nursing home residents and their relatives are neither simple nor are they neutral everyday objects. The gap between the recommended uses and the real uses (taking into account how these approaches were developed and embraced) gives rise to various configurations in which the tools’ mediating capacity is not a given but must be continuously (re-)created and (re-)established to enable the creation of a transitional relational field in the presence of the mediating object. This is essential to prevent studies of inequalities in the adoption of digital tools adopting a “*bio-social shunt*,” meaning the temptation to eliminate references to the unconscious by squeezing the latter between data borrowed from the fields of biology or physics and from socio-anthropological inspirations (Green, 1991, 175), in accordance with researchers’ interdisciplinary projects. The contributions of the research also seem to us to be essential to support the reflection, within nursing homes, on the different modalities of use of the digital technology and their effects, in order to allow the professionals to accompany the use of these technological devices in an aim which favors their subjective and positive appropriation by the older adults.

## Data availability statement

The raw data supporting the conclusions of this article will be made available by the authors, without undue reservation.

## Ethics statement

The studies involving human participants were reviewed and approved by the Research Ethics Committee of the University of Strasbourg, Strasbourg, France (accreditation number: Unistra/CER/2021–10). The patients/participants provided their written informed consent to participate in this study.

## Author contributions

CR participated in the conduct of the research, coordinated the writing of the manuscript, and drafted the psychological contributions to the manuscript. RM contributed to the analysis of the data, the discussion of the results, and the writing of the manuscript. CH was involved in conducting the research, analyzing the research results, and writing the manuscript. VB, FC, and CS participated in conducting the research and finalizing the manuscript. CL led the research, participated in conducting the research, in the analysis of the research results, and in the writing of the manuscript. All authors listed have made a substantial and intellectual contribution to the work, and approved it for publication.

## Funding

This work was carried out with the financial support of the Grand Est Region and the Ministry of Higher Education, Research and Innovation (France) via the National Research Agency (ANR), as part of the call for shared initiative projects “Grand Est Resilience” (ANR-20-GES1-0004).

## Conflict of interest

The authors declare that the research was conducted in the absence of any commercial or financial relationships that could be construed as a potential conflict of interest.

## Publisher’s note

All claims expressed in this article are solely those of the authors and do not necessarily represent those of their affiliated organizations, or those of the publisher, the editors and the reviewers. Any product that may be evaluated in this article, or claim that may be made by its manufacturer, is not guaranteed or endorsed by the publisher.
