# Supplementary material for: Analyzing the use of videoconference by and for older adults in nursing homes: an interdisciplinary approach to learn from the pandemic
Source: Front Psychol. 2023 May 5;14:1154657. doi: 10.3389/fpsyg.2023.1154657 (PMC10196051; doi:10.3389/fpsyg.2023.1154657)
Supplement: Supplementary file 1 [file Table_1.DOCX]

**Table 1_Summary table of data collection**

| **Researcher who conducted the interview** | **ID** | **Type**  **(person / place / link to others)** | **Duration** | **Age range** | **Genre** |
| --- | --- | --- | --- | --- | --- |
| **Exploratory interviews** | | | | | |
| Sociology researcher (interview 1) | A1 | Long-term care nurse | 60 min. | 40-45 | F |
| Sociology researcher (interview 2) | A2 | Management head_health and social care institute_people with disabilities | 50min. | 50-55 | H |
| Sociology researcher (interview 3) | A3 | Territorial Health Officer | 70 min. | 25-30 | F |
| Sociology researcher (interview 4) | A4 | Psychomotor therapist_Nursing Home | 60 min. | 30-35 | F |
| Sociology researcher (interview 5) | A5 | Management head_Nursing Home | 49 min. | 35-40 | H |
| Sociology researcher (interview 6) | A6 | Daughter_Resident (health and social care worker) | 210 min. | 60-65 | F |
| Sociology researcher (interview 7) | A7 | Medical-psychological assistant | 117min. | 60-65 | H |
| Researcher in management sciences (interview 1) | C1 | Management head_Nursing Home | 15 min. | 50-55 | H |
| Researcher in management sciences (interview 2) | C2 | Health and social care management head | 15 min. | 60-65 | H |
| Researcher in management sciences (interview 3) | C3 | Management head_Hospital | 45 min. | 50-55 | H |
| Researcher in management sciences (interview 4) | C4 | Nurse coordinator | 30 min. | 40-45 | F |
| Researcher in management sciences (interview 5) | C5 | Management head_Hospital_ Nursing Home | 70 min. | 50-55 | H |
| Researcher in management sciences (interview 6) | C6 | Bank management controller | 15 min. | 45-50 | F |
| Researcher in management sciences (interview 7) | C7 | Management head_ Nursing Home | 90 min. | 50-55 | F |
| Researcher in management sciences (interview 8) | C8 | Management head_ Nursing Home | 90 min. | 45-50 | H |
| Researcher in management sciences (interview 9) | C9 | Management head_ Nursing Home | 90 min. | 50-55 | H |
| Researcher in management sciences (interview 10) | C10 | Management head_ Nursing Home | 90 min. | 50-55 | H |
| Researcher in management sciences (interview 11) | C11 | Medical coordinator | 120 min. | 50-55 | F |
| Researcher in management sciences (interview 12) | C12 | Management head_ Nursing Home | 90 min. | 35-40 | F |
| Researcher in management sciences (interview 13) | C13 | Management head_ Nursing Home | 90 min. | 40-45 | F |
| Researcher in management sciences (interview 14) | C14 | Management head_ Nursing Home | 120 min. | 40-45 | H |
| Researcher in management sciences (interview 15) | C15 | Management head_ Nursing Home | 60 min. | 40-45 | H |
| Researcher in management sciences (interview 16) | C16 | Management head_ Nursing Home | 60 min. | 35-40 | F |
| **NURSING HOME 1 (peri-urban area)** | | | | | |
| Sociology researcher (interview 8) | A8 | Resident 1 (former housewife) | 70 min. | 80-85 | F |
| Sociology researcher (interview 9) | A9 | Resident 2 (former accountant) | 81 min. | 70-75 | F |
| Sociology researcher (interview 10) | A10 | Brother_Resident 2 (retired, former Management head of a medical-social institution) | 27 min. | 70-75 | H |
| Sociology researcher (interview 11) | A11 | Sun_Resident 3 (artist - author) | 56 min. | 30-35 | H |
| Sociology researcher (interview 12) | A12 | Daugther_Resident 3 (cultural sector worker) | 50 min. | 30-35 | F |
| Sociology researcher (interview 13) | A13 | Medical coordinator | 44min. | 45-50 | F |
| Sociology researcher (interview 14) | A14 | Reception officer | 52min. | 30-35 | F |
| Sociology researcher (interview 15) | A15 | Social Life Assistant | 130 min. | 55-60 | H |
| Sociology researcher (interview 16) | A16 | Social Life Assistant 1 (2^e^ étage) | 44 min. | 45-50 | F |
| Sociology researcher (interview 17) | A17 | Social Life Assistant 2 (2^e^ étage) | Prise de notes | 50-55 | F |
| Sociology researcher (interview 18) | A18 | Nursing assistant 3 (1e étage) | 50 min. | 55-60 | F |
| Sociology researcher (interview 19) | A19 | Nursing assistant 4 (rez-de-chaussée) | 48min. | 35-40 | F |
| Sociology researcher (interview 20) | A20 | Psychologist | 48 min. | 30-35 | F |
| Sociology researcher (interview 21) | A21 | Nurse | 49 min. | 45-50 | F |
| Sociology researcher / Researcher in management sciences (interview 22) | A22 | Management head_interview 1 | 25 min. | 60-65 | F |
| Psychology researcher (interview 1) | B1 | Resident 2 (former accountant) | 60 min. | 70-75 | F |
| Psychology researcher (interview 2) | B2 | Resident 1 (former housewife) | 75 min. | 80-85 | F |
| Psychology researcher (interview 3) | B3 | Psychologist | 84 min. | 30-35 | F |
| Psychology researcher (interview 4) | B4 | Social Life Assistant_ Resident 4 | 90 min. | 55-60 | H |
| Psychology researcher (interview 5) | B5 | Sister_ Resident 4 (retired) | 82 min. | 70-75 | F |
| Psychology researcher (interview 6) | B6 | Reception officer | 56 min. | 30-35 | F |
| Psychology researcher (interview 7) | B7 | Nurse | 37 min. | 45-50 | F |
| Psychology researcher (interview 8) | B8 | Daugther_Resident 3 (cultural sector worker) | 73 min. | 30-35 | F |
| Researcher in management sciences (interview 17) | C17 | Management head_interview_2 | 68 min. | 60-65 | F |
| Researcher in management sciences (interview 18) | C18 | Management head_interview_3 | 41 min. | 60-65 | F |
| **NURSING HOME 2 (peri-urban area)** | | | | | |
| Sociology researcher (interview 23) | A20 | Resident 1 (former seamstress) | 69 min. | 90-95 | F |
| Sociology researcher (interview 24) | A21 | Sun_Resident 1 (retired, former engineer) | 68 min. | 65-70 | H |
| Sociology researcher (interview 25) | A22 | Friend_Resident 1 (retired, former housewife) | 95 min. | 65-70 | F |
| Sociology researcher (interview 26) | A23 | Resident 2 (former shopkeeper) | 81 min. | 90-95 | F |
| Sociology researcher (interview 27) | A24 | Resident 3 | 29min. | 95-100 | F |
| Sociology researcher (interview 28) | A25 | Social coordinator | 50 min. | 45-50 | F |
| Sociology researcher (interview 29) | A26 | Nurse coordinator | 76 min. | 35-40 | H |
| Sociology researcher (interview 30) | A27 | Nurse | 19 min. | 50-55 | F |
| Sociology researcher (interview 31) | A28 | Psychologist | 106 min. | 60-65 | F |
| Sociology researcher (interview 32) | A29 | Logistics officer 1 | 22 min. | 45-50 | F |
| Sociology researcher (interview 33) | A30 | Civic service social animator | Prise de notes | 20-25 | F |
| Sociology researcher (interview 34) | A31 | Medical coordinator | 51 min. | 55-60 | H |
| Sociology researcher (interview 35) | A32 | Sun_Resident 4 (Retired, former sports teacher) | 59 min. | 60-65 | H |
| Sociology researcher (interview 36) | A33 | Management head_Nursing Home 2 | Prise de notes | 55-60 | F |
| Psychology researcher (interview 9) | B9 | Resident 4 (former housewife) | 76 min. | 90-95 | F |
| Psychology researcher (interview 10) | B10 | Resident 5 | 79 min. | 90-95 | F |
| Psychology researcher (interview 11) | B11 | Housekeeper | 55 min. | 50-55 | F |
| Psychology researcher (interview 12) | B12 | Social coordinator | 61 min. | 45-50 | F |
| Psychology researcher (interview 13) | B13 | Resident 1 | 57 min. | 90-95 | F |
| Psychology researcher (interview 14) | B14 | Sun_Resident 4 (retired, former sports teacher) | 76 min. | 60-65 | H |
| Psychology researcher (interview 15) | B15 | Sun_Resident 1 (retired, former engineer) | 61 min. | 65-70 | H |
| **NURSING HOME 3 (peri-urban area)** | | | | | |
| Researcher in management sciences (interview 19) | C19 | Management head | 50 min. | 40-45 | H |
| Researcher in management sciences (interview 20) | C20 | Nurse | 30 min. | 45-50 | F |
| Researcher in management sciences (interview 21) | C21 | Nurse coordinator | 45 min. | 45-50 | F |
| Researcher in management sciences (interview 22) | C22 | Collective interview :  Social coordinator +  Psychologist | 50 min. | 45-50  35-40 | F  F |
| Researcher in management sciences (interview 23) | C23 | Social coordinator_sport | 15 min. | 20-25 | F |
| Researcher in management sciences (interview 24) | C24 | Logistics officer | 15 min. | 40-45 | F |
| Researcher in management sciences (interview 25) | C25 | Skype_Family | 15 min. | 60-70 | H&F |
| Researcher in management sciences (interview 26) | C26 | Logistics officer | 15 min. | 25-30 | F |
| Researcher in management sciences (interview 27) | C27 | Resident 1 | 30 min. | 90-95 | F |
| Researcher in management sciences (interview 28) | C28 | Resident 2 | 30 min. | 85-90 | H |
| Researcher in management sciences (interview 29) | C29 | Medical coordinator | 30 min. | 45-50 | H |
| **NURSING HOME 4 (urban area)** | | | | | |
| Researcher in management sciences (interview 33) | C33 | Management head | 42 min. | 35-40 | H |
| Researcher in management sciences (interview 34) | C34 | Management head | 39 min. | 35-40 | H |
| **NURSING HOME 5 (rural area)** | | | | | |
| Researcher in management sciences (interview 30) | C30 | Collective interview:  Social coordinator,  Nurse coordinator,  Logistics officer | 137 min. | 40-45  45-50  45-50 | F  F  F |
| Researcher in management sciences (interview 31) | C31 | Nursing assistant | 48 min. | 35-40 | F |
| Researcher in management sciences (interview 32) | C32 | Management head | 84 min. | 40-45 | F |
| **NURSING HOME 6 (rural area)** | | | | | |
| Researcher in management sciences (interview 35) | C35 | Management head | 40 min. | 50-55 | F |
| Researcher in management sciences (interview 36) | C36 | Nurse coordinator | 62 min. | 35-40 | H |
| Researcher in management sciences (interview 37) | C37 | Social coordinator | 50 min. | 45-50 | F |
| Researcher in management sciences (interview 38) | C38 | Logistics officer (housekeeper) | 23 min. | 25-30 | F |

Total interviews : 89
