# Supplementary material for: Analyzing the use of videoconference by and for older adults in nursing homes: an interdisciplinary approach to learn from the pandemic
Source: Front Psychol. 2023 May 5;14:1154657. doi: 10.3389/fpsyg.2023.1154657 (PMC10196051; doi:10.3389/fpsyg.2023.1154657)
Supplement: Supplementary file 2 [file Table_2.DOCX]

**Table 2_Interview Guide for Nursing Homes Directors**

Questions related to nursing homes:

- Membership to a management body/Geographical area
- Number of beds / places / accommodation rates?
- Number of employees? Volunteers?
- Distribution of nurses, nursing assistants, facilitators /…?
- Dependence level (Pathos - Gir)?
- Any special information about the nursing home?

Respondent profile:

Job tenure, work experience, training, age?

The uses of digital devices (referred to as digital tools/ tablets /videoconferencing tools such as Skype, Zoom, and WhatsApp depending on the case):

1. **Today**

Do you use tablets or any other digital tools with residents?

If so, which ones? How often? With what objective in mind?

If not, does anyone else in the nursing home use these tools with residents?

If not, why?

Do residents use them alone? If so, which tools? How often?

1. **Feedback on the time frame**

Has there been any change in usage? Especially during lockdown?

What tools were used? By whom? With what objective in mind?

Have there been any changes, phases? If so, which ones?

1. **Nature and strength of ties**

With whom were the video-calls organized?

How did the residents feel about these calls?

What do these calls allow or what did they allow? What didn’t they allow?

1. **Other ties**

Were there other means of communication during these periods of restricted access?

1. **Key factors for success or failure (with emphasis on organizational and managerial dimensions)**

Which challenges did you face while using tablets?

What are the conditions for a successful video-call? What are the barriers to the use of tablets? What are the obstacles to video-calls with / by / for residents?

1. **Impact of the use of digital tools in nursing homes**

Has the use of digital tools changed anything in the nursing home, for instance the organization or management?
