# Supplementary material for: Analyzing the use of videoconference by and for older adults in nursing homes: an interdisciplinary approach to learn from the pandemic
Source: Front Psychol. 2023 May 5;14:1154657. doi: 10.3389/fpsyg.2023.1154657 (PMC10196051; doi:10.3389/fpsyg.2023.1154657)
Supplement: Supplementary file 3 [file Table_3.DOC]

**Table 3_Interview Guide for Residents**

1. How old are you?
2. When did you come to the nursing home?
3. How many children do you have? Can you tell me more (family history)?
4. Have you ever been married? (for how long, brief life history)
5. What was your profession before retirement?
6. Marital status
7. What type of home did you have? (house, appartement)
8. Would you say you feel at home here?
9. Did you encounter any difficulties when you were still living at home?
10. Who assisted you (professionals, relatives)?
11. Do the relatives who assisted you visit regularly (before – during – after COVID)
12. How did you experience the lockdown?
13. Can you describe your experience with videoconferencing?
14. Did you remain in contact with your loved ones over the phone?
15. Do you still do video-calls today? If not, why?
16. What do you think is the difference between face-to-face exchanges and video-calls?
17. What is your view of social ties? Of social ties in nursing homes?
