# Supplementary material for: Analyzing the use of videoconference by and for older adults in nursing homes: an interdisciplinary approach to learn from the pandemic
Source: Front Psychol. 2023 May 5;14:1154657. doi: 10.3389/fpsyg.2023.1154657 (PMC10196051; doi:10.3389/fpsyg.2023.1154657)
Supplement: Supplementary file 4 [file Table_4.DOCX]

**Table 4_Interview Guide for Professionals and Relatives**

*We are seeking testimony from residents, relatives and professionals who have experienced visitation restrictions in nursing homes and the implementation of digital tools seeking to promote social ties during this particular period.*

1. Can you tell me about how you felt about this particular period?
2. How did you experience lockdown?

What were your concerns?

Which resources did you know or think that you could count on?

1. How did you experience the implementation of digital video calling tools?

How did you participate in this implementation?

What were your objectives/expectations when these tools were implemented? What do you think were the goals/expectations of others? (other professionals, relatives, residents)

How were these tools introduced? In continuity or discontinuity (break/novelty) with previous professional/personal practices?

How have they influenced professional/personal habits? And your own practice/habits?

How was the reflection on the modalities of introduction of these tools undertaken (individual, group, institutional)?

How were these objectives and/or modalities likely to be shared and/or to bring about conflict?

What were your feelings, your encounter with these experiences?

What do you think are the favorable conditions/measures among residents/professionals/relatives for this tool to be beneficial? Could you give me an example or tell me about a successful implementation experience?

What do you think are the unfavorable conditions/measures for residents/professionals/relatives to use this tool? Could you give me an example/tell me about an unsuccessful implementation experience?

What were their roles?

In what ways were these tools used (or not)? On what initiative/based on what request?

Who have these tools benefited? Why? How? (guided to speak about targeted situations, regarding the residents met in particular)

1. What is your view of social ties in nursing homes?

How do they differ from other ties?

On what, on whom, are social ties based in this nursing home?

How are they revealed?

Which actors are involved?

What institutional support can/should they rely on?

Would you say that video-calls have helped or failed to promote social ties in this institution?

What discrepancies have you noticed between expectations and experiences?

What new demands/needs, if any, have emerged from these new arrangements?

1. What do you think will be the next steps after the implementation of these tools in this institution?
